# Transcatheter Aortic Valve Replacement Is Associated with Less Oxidative Stress and Faster Recovery of Antioxidant Capacity than Surgical Aortic Valve Replacement

**DOI:** 10.3390/jcm8091364

**Published:** 2019-09-02

**Authors:** Anna Komosa, Bartłomiej Perek, Piotr Rzymski, Maciej Lesiak, Jolanta M. Siller-Matula, Marek Grygier, Mateusz Puślecki, Marcin Misterski, Anna Olasińska-Wiśniewska, Mariola Ropacka-Lesiak, Zbigniew Krasiński, Przemysław Niedzielski, Tatiana Mularek-Kubzdela, Barbara Poniedziałek

**Affiliations:** 1 1st Department of Cardiology, Poznan University of Medical Sciences, 61-848 Poznan, Poland; 2 Department of Cardiac Surgery and Transplantology, Poznan University of Medical Sciences, 61-848 Poznan, Poland; 3 Department of Environmental Medicine, Poznan University of Medical Sciences, 61-848 Poznan, Poland; 4 Department of Cardiology, Medical University of Vienna, A-1090 Vienna, Austria; 5 Department of Perinatology and Gynecology, Poznan University of Medical Sciences, 60-535 Poznan, Poland; 6 General and Vascular Surgery Institute, Poznan University of Medical Sciences, 61-848 Poznan, Poland; 7 Department of Analytical Chemistry, Adam Mickiewicz University, 61-614 Poznan, Poland

**Keywords:** surgical aortic valve replacement, transcatheter aortic valve implantation, oxidative stress, total antioxidant capacity

## Abstract

The objective of this study was to compare oxidative stress indices in 24 patients (mean ± SD age 71 ± 13 years) undergoing surgical aortic valve replacement (SAVR) versus transcatheter aortic valve replacement (TAVR). Serum total antioxidant capacity (TAC), copper/zinc ratio (Cu/Zn), activity of lactate dehydrogenase (LDH), and thiobarbituric acid reactive substances (TBARS) were assessed at four different time-points: pre-procedure, immediately post-procedure, and one day and two days after the procedure. All oxidative stress parameters were comparable in both groups pre-procedure. TAC decreased significantly when assessed immediately after procedures in both groups (*p* < 0.001); however, the magnitude of the reduction was more pronounced after SAVR (88% decrease from baseline: 1.8 ± 0.1 vs. 0.2 ± 0.03 mM) compared to TAVR procedures (53% decrease from baseline: 1.9 ± 0.1 vs. 1.0 ± 0.1 mM; *p* < 0.001). TAC returned to baseline two days after TAVR in all patients, but was still reduced by 55% two days after SAVR. In concordance, TBARS levels and Cu/Zn ratio increased significantly with maximum levels immediately after procedures in both groups (*p* < 0.001), but the magnitude of the increase was significantly higher in SAVR compared to TAVR (TBARS: 3.93 ± 0.61 µM vs. 1.25 ± 0.30 µM, *p* = 0.015; Cu/Zn ratio: 2.33 ± 0.11 vs. 1.80 ± 0.12; *p* < 0.001). Two days after the procedure, TBARS levels and the Cu/Zn ratio returned to baseline after TAVR, with no full recovery after SAVR. TAVR is associated with a lesser redox imbalance and faster recovery of antioxidant capacity compared to SAVR.

## 1. Introduction

Aging of societies, prolonged expected life span, and substantial progress in medicine in developed countries has resulted in the predominance of degenerative aortic stenosis (AS) among cardiac valvular pathologies requiring invasive treatment [1,2]. Currently, AS is treated either by cardiac surgeons or interventional cardiologists. Although surgical aortic valve replacement (SAVR) with the use of cardio-pulmonary bypass (CBP) remains the standard of severe AS treatment, at present, elderly and intermediate to high-risk patients undergo minimally invasive transcatheter aortic valve replacement (TAVR) with a trend toward low-risk populations [3,4,5]. In the last few years, the expectations of patients undergoing invasive cardiac procedures, including aortic valve surgery have been markedly increased. Thus, the optimal therapeutic option should not only be the most efficient but also the safest one. TAVR procedures as a minimally invasive option appear to be safer compared to SAVR [4]. One can expect that a choice of therapy for symptomatic severe aortic stenosis may have an impact on the magnitude of post-surgical oxidative stress and eventually on early and late outcomes. There are several factors that may predict unfavorable outcomes and postoperative adverse events.

These include systemic oxidative stress, triggered by the release of reactive oxygen species (ROS) above antioxidant capacity, as a response to intraoperative trauma. It has been established that oxidative stress plays a crucial role in the development and perpetuation of inflammation, which can alter the post-surgical recovery process [5]. It has also been found to be involved in the pathogenesis of postoperative atrial fibrillation, and irrespective to the type of surgery, it may increase mortality and morbidity [6,7,8]. Oxidative stress is among factors predisposing to acute kidney injury following cardiac surgical procedures [9]. It is also plausible that it plays a crucial role in cardiac surgery-associated multi-organ dysfunction [10].

A variety of biomarkers of redox balance have been studied and applied in various clinical and experimental scenarios. The generated ROS have extremely low stability, and, thus, their measurement in clinical practice is largely limited. Therefore, the recommended approach is to use a battery of complementary parameters to characterize the potential outcomes of oxidative stress induced by surgical procedures such as the level of lipid peroxidation, total antioxidant capacity (TAC), activity of lactate dehydrogenase (LDH), and copper/zinc (Cu/Zn) ratio in serum of patients [11,12]. Cu and Zn have pro-oxidant and antioxidant properties, respectively, so that an increase in their ratio is expected to condition redox imbalance, and has been associated with systemic oxidative stress [13]. This phenomenon can, inter alia, trigger lipid peroxidation, a chain reaction initiated by the hydrogen abstraction or the addition of an oxygen radical, generating predominantly genotoxic malondialdehyde and resulting in the oxidative damage of polyunsaturated fatty acids. Under such conditions, a significant decrease of antioxidant capacities and cell membrane damage can be expected [14,15]. The loss of its integrity can, in turn, be measured by means of the LDH released into extracellular space [16].

As the comparative data concerning the oxidative stress between both procedures is limited, the aim of our study was to compare serum oxidative stress indices (total antioxidant capacity, thiobarbituric acid reactive substances, copper/zinc ratio, and total lactate dehydrogenase activity) in patients undergoing SAVR vs. TAVR procedures during the hospital stay.

## 2. Materials and Methods

### 2.1. Patients

We investigated oxidative stress indices in 24 consecutive patients (14 men and 10 women) with a mean age (± SD) of 71 ± 13 years who underwent elective SAVR (*n* = 12) or TAVR (*n* = 12) procedures (Table 1) between May 2016 and March 2017. All of the studied individuals satisfied the criteria of high-gradient aortic stenosis defined according to valid ESC guidelines [17]. Baseline laboratory results are summarized in Table 2.

All patients were asked to give their written informed consent prior to participating in the study. The protocol of this trial and the informed consent were approved by the Ethical Committee of the Medical University in Poznan (Approval No. 968/15, Date of approval: 5 November 2015).

### 2.2. Surgical Procedure (SAVR)

All operations were performed from full median sternotomy with the use of cardio-pulmonary bypass (CPB) in moderate hypothermia (28 °C) and cardioplegic cardiac arrest according to St Thomas Hospital II formula [18]. CPB was conducted through an arterial cannula introduced to the ascending aorta and two-staged venous one to the right atrium. After the ascending aorta was opened, the aortic valve was completely removed and an aortic prosthesis using 2-0 sutures with Teflon pledges was implanted. After the aortotomy was closed with a 5-0 monofilament suture and de-airing of the left heart was completed, the ascending aorta was de-clamped and the reperfusion phase of CPB initiated. Successful weaning from CPB was followed by removal of all cannulas, protamine administration, careful hemostasis, and closure of the chest.

### 2.3. Percutaneous Aortic Valve Implantation (TAVR)

Patients were eligible for TAVR on the basis of the institutional heart team’s decision (interventional cardiologist, cardiac surgeon, and echocardiography specialist).

The pre-procedural evaluation included: coronary angiography; transthoracic echocardiography (TTE) and transesophageal echocardiography (TEE); contrast-enhanced computed tomography with off-line reconstructions to evaluate the aorta, femoral, and iliac arteries. The final decision regarding the way of vascular approach was made based on the results of a CT scan.

General anesthesia or deep sedation was used during the procedures. The TTE monitoring was performed and a temporary pacemaker was inserted from the jugular vein for rapid pacing and as prevention of iatrogenic atrioventricular block consequences [19].

In patients with a percutaneous femoral approach, ProStar™ (Abbott Vascular, Redwood City, CA, USA) system or two Perclose ProGlide™ devices (Abbott Vascular Devices, Redwood City, CA, USA) were introduced before insertion of the vascular sheath. The Medtronic CoreValve Evolut R prosthesis was implanted in all cases. Once the prosthesis was correctly positioned, expanded, and deployed, the contrast injection was performed to assess the presence and degree of paravalvular leak (PVL). Control angiography of the access site was performed to assess vessel patency and possible bleeding [20].

### 2.4. Serum Collection

Serum samples were collected by centrifugation from the whole blood at 4 different time points: pre-procedure, immediately post-procedure, and one day and two days after the procedure. The following parameters were assessed in patients’ serum: TAC, lipid peroxidation, LDH activity, and Cu/Zn ratio.

### 2.5. Determination of Total Antioxidant Capacity

The total antioxidant capacity (TAC) of serum AC was evaluated according to method by Rice-Evans and Miller [21]. Briefly, it is based on the inhibition of the radical cation of 2,2’-azino-bis (3-ethylbenzothiazoline 6-sulphonate), (ABTS). The ABTS cation is formed by the interaction of 150 µM of ABTS with the ferrylmyoglobin radical species, produced by the activation of 2.5 µM of metmyoglobin with 75 µM of hydrogen peroxide. The antioxidative activity results in suppression of the absorbance (734 nm) of the ABTS radical cation. After the addition of ABTS and myoglobin to serum sample, the reaction was initiated with hydrogen peroxide. Following the incubation for 5 min at 21°C, the absorbance of the product was read and compared to a calibration curve (*r*^2^ = 0.98) prepared using the 0.5–2.0 mM of 6-hydroxy-2,5,7,8-166 tetramethylchroman-2-carboxylic acid (Trolox) (Sigma-Aldrich, St. Louis, MO, USA), a water soluble analogue of vitamin E. The final results were calculated as mM Trolox equivalents. Each sample was analysed in triplicate.

### 2.6. Determination of Lipid Peroxidation

The level of lipid peroxidation was assessed by measuring the concentration of thiobarbituric acid reactive substances (TBARS), which are a mixture of aldehydes, predominantly represented by malondialdehyde (MDA). To this end, an adduct of MDA and thiobarbituric acid (TBA) was generated by mixing 100 μL of serum samples with 100 μL of 10% trichloroacetic acid and 800 μL of TBA. The reaction was carried out at 95 °C for 60 min, and then inhibited by placing on an ice bath for 10 min, and eventually centrifuged at 4 °C (1600× *g*, 10 min). The final product was measured fluorometrically at the excitation/emission wavelengths of 535/550 nm. The obtained values were compared to a calibration curve of the MDA standard (0.0–50.0 μM; *r*^2^ = 0.99) (Cayman Chemical, Ann Arbor, MI, USA) and given as µM. Each sample was analysed in triplicate.

### 2.7. Determination of the Cu/Zn Ratio

Serum samples (1.0 mL) were digested with 3 mL of HNO_3_ in closed Teflon vessels using the microwave sample digestion system Mars 6 (CEM, USA) by ramping to 180 °C for 20 min and holding for 30 min. The samples were then diluted to a 5.00 mL with ultrapure MilliQ water (Millipore, Burlington, MA, USA). The concentration of Cu and Zn was evaluated with an inductively coupled plasma optical emission spectrometer Agilent 5110 ICP-OES (Agilent, Palo Alto, CA, USA). The following common instrumental parameters were used for determination of all elements: RF power 1.2 kW, plasma gas (argon) flow 12 L·min^–1^, nebulizer gas (argon) flow 0.7 L·min^–1^, axial plasma observation. The instrument was calibrated with CM17 PrimAg Plus and KP7 PrimAg (Romil, Cambridge, UK) analytical standards. A certified material ERM-DA120 (human serum, LGC Standards, Teddington, UK) was used for validation. The following wavelengths (nm) were applied: Cu—327.395 and Zn—213.857. The Cu/Zn ratio was calculated from obtained serum concentration of each element.

### 2.8. Determination of Total Activity of Lactate Dehydrogenase

The activity of total LDH was evaluated with a Lactate Dehydrogenase Activity Assay Kit (Sigma-Aldrich, Saint Louis, MO, USA). The assay is based on principle that LDH catalyzes the reduction of NAD to NADH, and the latter reacts with the provided probe to generate a product which can be detected spectrophotometrically at 450 nm. The obtained values are compared to a standard curve prepared with NADH standard (*r*^2^ = 0.99) and given as U L^−1^ (the amount of LDH needed to catalyze the conversion of lactate into pyruvate to generate 1.0 µmol of NADH per min at 37°C). Each sample was analysed in triplicate. 

### 2.9. Statistical Analysis

All statistical analyses were performed using Statistica 10.0 for Windows software (StatSoft, Inc., Tulsa, OK, USA). Based on the assumption that the nadir level of TAC in the SAVR will be at least 3-fold lower than in the TAVR group, we calculated that with a sample size of 12 per group, the study will have a 99% power to show a difference between groups with a two-sided *p* < 0.05. The Gaussian distribution of data was assessed with the Shapiro–Wilk test. Normally distributed data were presented as the means ± standard deviation (SD) and compared using unpaired T student test, whereas the variables that did not meet the normality assumption were presented as median and interquartile range and compared using the Mann Whitney U test. The categorical variables were compared with Pearson’s chi-square test. The correlation coefficients between two independent variables in every study point were measured by means of the Spearman correlation coefficient. A *p*-value < 0.05 was considered statistically significant.

## 3. Results

### 3.1. Total Antioxidant Capacity (TAC)

**Figure 1:**
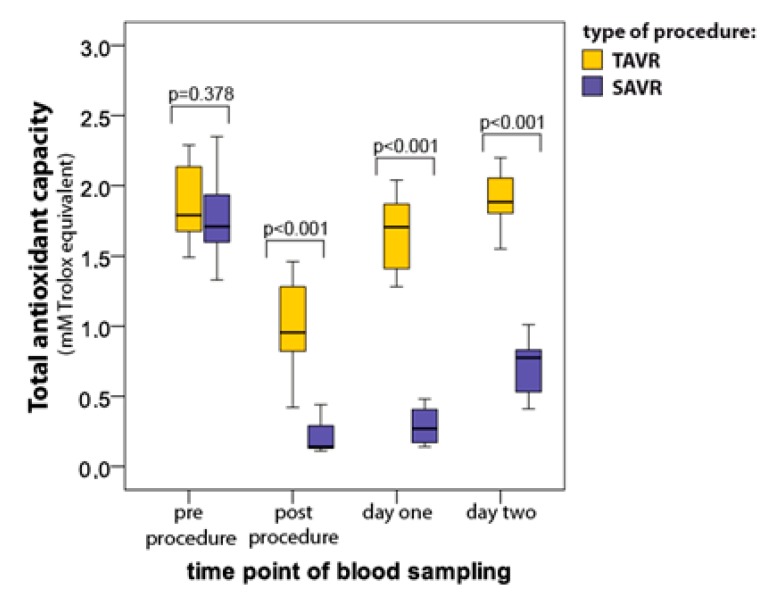
Serum total antioxidant capacity (median with interquartile range) in patients undergoing percutaneous aortic valve implantation (TAVR) (*n* = 12) and surgical aortic valve replacement (SAVR) (*n* = 12) procedures.

TAC decreased significantly when assessed immediately after procedures in both groups (*p* < 0.001), however TAC reduction was less pronounced in TAVR (median 1.79 ± interquartile range 0.48 vs. 0.96 ± 0.52 mM) as compared to SAVR (1.71 ± 0.34 vs. 0.14 ± 0.16 mM) (Figure 1). TAC returned to baseline two days after TAVR (1.88 ± 0.30 mM) but was still reduced by 56% two days after SAVR (0.78 ± 0.35 mM; *p* = 0.003 vs. baseline; Figure 1).

### 3.2. Thiobarbituric Acid Reactive Substances (TBARS)

**Figure 2:**
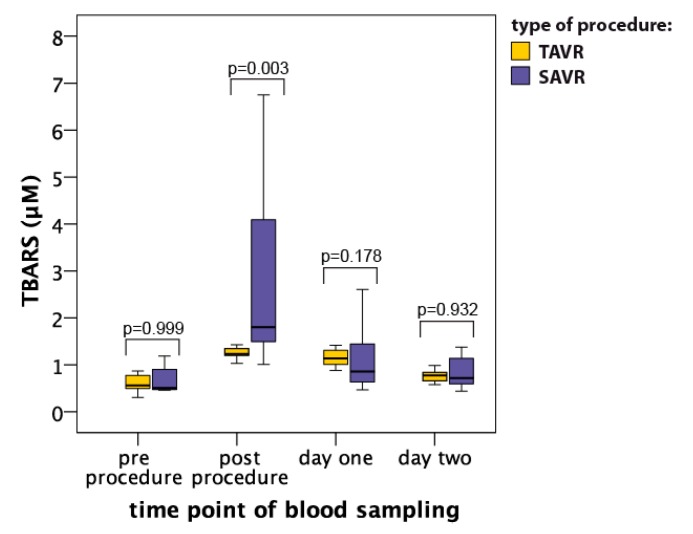
Serum thiobarbituric acid reactive substances (TBARS) level (median with interquartile range) in patients undergoing TAVR (*n* = 12) and SAVR (*n* = 12) procedures.

The level of lipid peroxidation assessed by means of concentrations of thiobarbituric acid reactive substances (TBARS) increased significantly in both groups soon after the procedures and the maximal levels, reached soon after the procedures, were higher in the SAVR group as compared to the TAVR group (1.24 ± 0.15 μM vs. 2.56 ± 2.78 μM, respectively; *p* = 0.003; Figure 2). TBARS concentration dropped markedly already at day one after the procedures and was comparable between both groups (*p* = 0.178; Figure 2).

### 3.3. Copper/Zinc Ratio (Cu/Zn)

**Figure 3:**
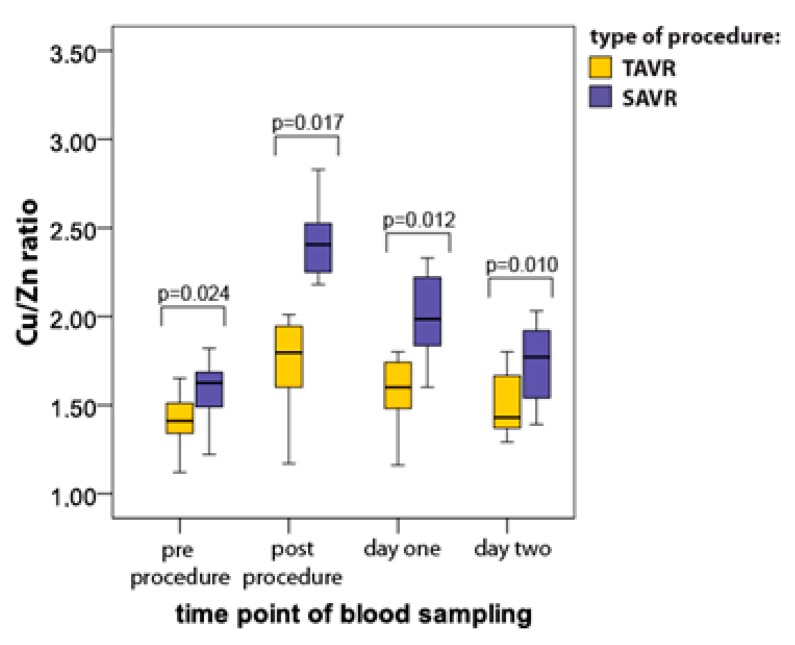
Serum Cu/Zn ratio (median with interquartile range) in patients undergoing TAVR (*n* = 12) and SAVR (*n* = 12) procedures.

Cu/Zn ratio was slightly higher in the SAVR group as compared to the TAVR group already at baseline (Figure 3). The significant increase in the Cu/Zn ratio was found soon after surgery in both groups; however, the maximal levels were significantly higher after SAVR vs. TAVR (SAVR 2.33 ± 0.11 vs. TAVR 1.80 ± 0.12, respectively; *p* = 0.017) (Figure 3). The differences between the groups remained significant throughout the whole study period (*p* = 0.010; Figure 3). Interestingly, 48 h after procedures the Cu/Zn ratio reached baseline value but only in the TAVR subgroup (baseline 1.41 ± 0.21 vs. 48 h after procedure 1.43 ± 0.31; *p* > 0.05).

### 3.4. Total Lactate Dehydrogenase Activity (LDH)

**Figure 4:**
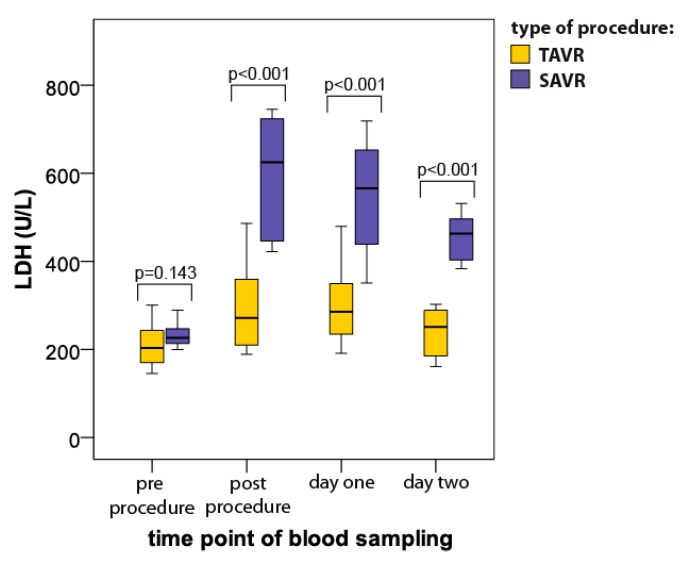
Serum lactate dehydrogenase (LDH) activity level (median with interquartile range) in patients undergoing TAVR (*n* = 12) and SAVR (*n* = 12) procedures.

LDH activity increased significantly after the procedures in both groups. However, the time course of this increase presented different patterns in the blood samples harvested from SAVR vs. TAVR patients (Figure 4). The post-procedure values of LDH were significantly higher in the SAVR group (625 ± 278 IU/L) compared to the TAVR group (289 ± 158 IU/L; *p* < 0.001; Figure 4) and the difference between the groups remained statistically significant during the whole study period (Figure 4). Two days after the procedure, the LDH level returned to baseline in the TAVR group but was still increased by more than 95% in the SAVR group (baseline: 233 ± 39 IU/L vs. day 2: 463 ± 99 IU/L; *p* = 0.002).

### 3.5. Relationship Between Oxidative Stress Markers

**Figure 5:**
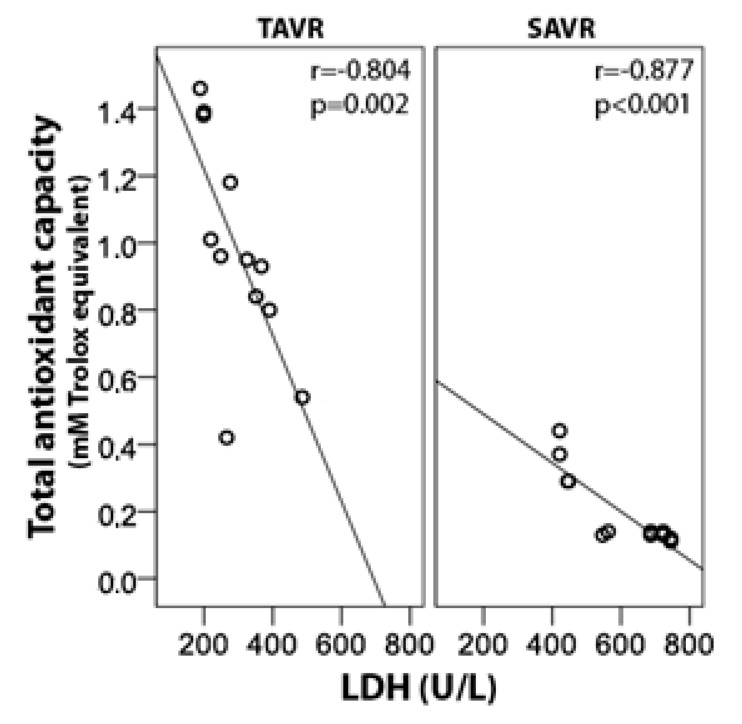
Correlation between serum total antioxidant capacity and LDH immediately after TAVR and SAVR procedures.

All TAC values calculated after either TAVR or SAVR procedures correlated strongly and negatively with both LDH activity (*r* = −0.70) and Cu/Zn ratio (*r* = −0.64). Additionally, a strong positive relationship between LDH activity and Cu/Zn ratio was also noted (*r* = 0.54). The detailed correlation coefficients soon after procedures completion are outlined in Table 3 and one example is presented graphically as Figure 5. Similar findings in both groups were seen. No association between TBARS concentrations and other parameters of oxidative stress were found.

## 4. Discussion

In recent years, TAVR has become a more common therapeutic option applied for the efficient treatment of patients with severe and symptomatic AS. As a minimally invasive procedure without the necessity to connect subjects to CPB it enabled the treatment of AS patients that previously had not been eligible for conventional SAVR [22]. Additionally, promising early outcomes of high-risk SA individuals together with increased operator experience and improved device systems have led to the extension of this technology to the others, including intermediate- and even low-risk individuals [23,24].

There is growing evidence that TAVR may be competitive with SAVR in terms of faster recovery (shorter Intensive Care Unit (ICU) stay) and reduced morbidity [25,26]. This phenomenon may be explained by its less-invasive procedural/surgical approach (shorter skin incisions). However, this does not answer many questions regarding the higher organ dysfunction rate following SAVR comparing to TAVR. On the other hand, it is known that post-operative oxidative stress and inflammation (which can be initiated by a former), positively related to the invasiveness and damaging nature of procedures, may have a prognostic value in unfavorable outcomes of surgical procedure, and postoperative adverse events. The present study clearly highlights that TAVR induces significantly lower oxidative stress as measured by means of different, complementary biomarkers. Our study, although employing different markers of oxidative stress, also supports earlier observations in which patients undergoing TAVR were not reported to experience changes in the static oxidation-reduction potential and reduced glutathione [27,28]. It can, thus, be hypothesized that favorable outcomes following TAVR may at least partially result from a lower generation of oxidative agents and reduced systemic redox imbalance as compared to SAVR.

The novelty of our study is the observation that although there is an imbalance in redox state following TAVR, it appears to be temporary and returns to baseline status very quickly usually within 48 h following these procedures—which is not a case in SAVR subjects.

Moreover, our study has additional practical value. We have shown, for the first time, markers of redox state that are not usually correlated significantly with LDH activity, a conventional biochemical parameter. Thus, its routine measurements after procedures on the aortic valves, either SAVR or TAVR, may potentially help to identify patients with a significantly disturbed redox state and simultaneously at high risk of postprocedural organ failure. Physicians taking care of these patients at ICUs have enough time to take appropriate preventative measures as the aforementioned markers are very early (usually a few minutes after AVR).

ROS (reactive oxygen species) include oxygen ions, free radicals, and peroxides and represent the products of normal oxygen-consuming metabolic processes. Most intracellular ROS are derived from superoxide, which is generated by the one-electron reduction of O2, mostly at various sites in mitochondria [29]. An imbalance between ROS generation and the ability of the biological system to detoxify the reactive intermediates or to repair the resulting damage is known as an oxidative stress [30]. We want to highlight the important role of oxidative stress in platelet function and the potential importance of the assessment of platelet redox state in clinical research and practice. There are various biochemical and analytical methods that can be used in the evaluation of the redox state in platelets of patients who undergo medical interventions [31]. We found experimental and clinical studies that have shown a correlation between the level of oxidative stress markers and heart failure, myocardial ischemia, and various forms of cardiomyopathies [32,33,34]. There are only a few investigations, however, reporting on the subject of Cu/Zn ratio changes in patients with cardiovascular diseases. We demonstrate that the copper/zinc (Cu/Zn) ratio is a marker of cellular homeostasis disruption. In age-related degenerative diseases the Cu/Zn ratio was significantly and positively related to systemic oxidative stress status. A variation of the ratio due to the serum zinc concentration, which in ischemic heart disease presents values over the upper range and in acute myocardial infarction decreases below the lower cut-off value, has also been proven [35]. Clarification of these mechanisms may lead to novel therapeutic strategies. Further investigations are required to assess whether the oxidative stress markers have any impact on long-term outcome of patients who undergo TAVR or SAVR.

However, the present study provides valuable information on TAVR and SAVR outcome in view of systemic oxidative stress, one should acknowledge its limitations. Firstly, the study encompassed a small sample size due to the complex protocol including sophisticated surgical procedures preceded by many preparations, appropriate timing of blood collection (at four exactly defined time-points), correct sample transfer to the laboratory in a different location than the hospital, and eventually time-consuming and costly biochemical investigations. Moreover, there was a significant difference in the ages of patients undergoing TAVR and SAVR procedures (mean 80 vs. 63 years, respectively). This is due to valid clinical recommendations regarding management with aortic stenosis. TAVR is a method of choice in the treatment of elderly and high-risk subjects while SAVR is for relatively young and healthy individuals. One should, however, note that with the exception of Cu/Zn ration, the baseline parameters of oxidative stress in both studied groups of patients did not differ significantly.

## 5. Conclusions

This study compared oxidative stress markers in patients undergoing surgical aortic valve replacement SAVR vs. TAVR. As demonstrated, both procedures affected serum TAC, TBARS, Cu/Zn ratio, and LDH activity, with maximum levels being reached immediately after procedures. The magnitude of increase was higher after SAVR, and, in contrast to TAVR, no full recovery was seen after this procedure. The study indicates that redox imbalance following TAVR is only temporary and highlights its advantageous over SAVR.

## Figures and Tables

**Table 1:** Baseline characteristics of studied patients (*n* = 24).

Variable	TAVR (*n* = 12)	SAVR (*n* = 12)	*p* Value
Gender (Male)	6 (50%)	7 (58%)	0.70
Age (years)	80 (± 3)	63(± 10)	0.0006
Weight (kg)	1.64 (± 0.06)	1.67 (± 0.11)	0.43
Height (m)	74.2 (± 12.3)	78.8 (± 13.2)	0.39
BMI (kg/m^2^)	27.5 (± 4.7)	28.2 (± 3.9)	0.73
Obesity (BMI > 30 kg/m^2^)	5 (42%)	4 (33%)	0.69
Systemic hypertension	5 (42%)	8 (67%)	0.24
Diabetes mellitus	6 (50%)	3 (34%)	0.43
Prior PCI	5 (42%)	3 (25%)	0.41
Prior MI	3 (25%)	0	0.08
Prior stroke/TIA	2 (17%)	0	0.17
Prior CABG	2 (17%)	1 (8%)	0.56
COPD	2 (17%)	1 (8%)	0.56
Atrial fibrillation	3 (25%)	2 (17%)	0.63

CABG—coronary artery bypass grafting, PCI—percutaneous coronary intervention, MI—myocardial infarction, TIA—transient ischemic attack, COPD—chronic obstructive airway disease, BMI—body mass index.

**Table 2:** Baseline laboratory results.

Variable	TAVR (*n* = 12)	SAVR (*n* = 12)	*p* Value
WBC (10 × 9/L)	7.5 (± 2.0)	7.7(± 1.9)	0.20
HGB (mmol/L)	8.2 (± 0.9)	8.6 (± 0.9)	0.30
RBC (10 × 12/L)	4.2 (± 0.4)	4,5 (± 0.5)	0.14
HCT (L/L)	0.40 (± 0.04)	0.41 (± 0.04)	0.56
PLT (10 × 9/L)	215 (± 115)	207 (± 86)	0.81
CREA (µmol/L)	98 (± 23)	82 (± 22)	0.08
eGFR	64.4 (± 15.5)	80.7 (± 21.3)	0.02
ESR	18.3 (± 15.3)	11.8 (± 13.8)	0.27

WBC—white blood count, HGB—hemoglobin, RBC—red blood count, HCT—hematocrit, PLT—platelets, CREA—serum creatinine, eGFRestimated—gromerular filtration rate (MDRD), ESR—erythrocyte sedimentation rate.

**Table 3:** Correlation coefficients (*r* values) between examined oxidative stress markers assessed post-procedures (the first post-procedural sampling).

	TAC	LDH	Cu/Zn Ratio
**TAVR**			
**TAC**		−0.80	−0.73
**LDH**	−0.80		0.57
**Cu/Zn ratio**	−0.73	0.57	
**SAVR**			
**TAC**		−0.88	−0.61
**LDH**	−0.87		0.66
**Cu/Zn ratio**	−0.61	0.66	

Cu/Zn—copper/zinc, LDH—lactate dehydrogenase, SAVR—surgical aortic valve replacement, TAC—total antioxidant capacity, TAVR—transcatheter aortic valve replacement.
